# Exclusive breastfeeding continuation and associated factors among employed women in North Ethiopia: A cross-sectional study

**DOI:** 10.1371/journal.pone.0252445

**Published:** 2021-07-29

**Authors:** Kahsu Gebrekidan, Helen Hall, Virginia Plummer, Ensieh Fooladi

**Affiliations:** 1 Monash Nursing and Midwifery, Monash University, Frankston, Victoria, Australia; 2 Mekelle University, College of Health Sciences, School of Nursing, Mekelle, Ethiopia; 3 Federation University Australia, School of Health, Berwick, Australia; 4 Monash Nursing and Midwifery, Monash University, Clayton, Victoria, Australia; University of Mississippi Medical Center, UNITED STATES

## Abstract

**Background:**

Exclusive Breastfeeding (EBF) can prevent up to 13% of under-five mortality in developing countries. In Sub-Saharan Africa the rate of EBF at six months remains very low at 36%. Different types of factors such as maternal, family and work-related factors are responsible for the low rate of EBF among employed women. This study aimed to assess the prevalence of EBF continuation and associated factors among employed women in North Ethiopia.

**Materials and methods:**

A community-based, cross-sectional study was conducted in two towns of Tigray region, North Ethiopia. Employed women who had children between six months and two years were surveyed using multistage, convenience sampling. Women filled in a paper based validated questionnaire adopted from the Breastfeeding and Employment Study toolkit (BESt). The questions were grouped into four parts of sociodemographic characteristics, maternal characteristics, family support and work-related factors. Factors associated with EBF continuation as a binary outcome (yes/no) were determined using multivariable logistic regression.

**Results:**

Four-hundred and forty-nine women participated in this study with a mean (SD) age 30.4 (4.2) years. Two hundred and fifty-four (56.4%) participants exclusively breastfed their children for six months or more. The main reason for discontinuation of EBF was the requirement of women to return to paid employment (31.5%). Four-hundred and forty (98.2%) participants believed that breastfeeding has benefits either to the infant or to the mother. Three hundred and seventy-one (82.8%) of the participants received support from their family at home to assist with EBF, most commonly from their husbands and mothers. Having family support (adjusted odds ratio [AOR] = 2.1, 95%, CI 1.2–3.6; P = 0.005), having frequent breaks at work (AOR = 2.6, 95% CI, 1.4–4.8; P = 0.002) and the possibility of buying or borrowing required equipment for expressing breast milk (AOR = 1.7, 95% CI, 1.0–3.0; P = 0.033) were statistically associated with an increased chance of EBF.

**Conclusion:**

Although returning to work was reported by the study participants as the main reason for discontinuation of EBF, families and managers’ support play significant roles in EBF continuation, which in the absence of six-month’s maternity leave for employed women in Ethiopia would be of benefit to both mothers and children.

## 1. Introduction

In 1990 the World Health Organization (WHO) and United Nations International Children’s Emergency Fund (UNICEF) adopted a declaration on protection, promotion and support of breastfeeding, focused on the importance of exclusive breastfeeding (EBF) for at least six months [1]. Appropriate EBF practice can prevent up to 13% of under-five mortality in developing countries [2]. In light of this trend, the UNICEF and WHO set a target to increase the rate of EBF to 50% by 2025 [3, 4]. However, the rate of EBF for six months is suboptimal in many parts of the world [5]. In Sub-Saharan Africa, for example, only 36% mothers exclusively breastfeed their infants until six months [6]. In Ethiopia, the rate of EBF among employed women remains suboptimal. In Ethiopian studies conducted in Gondar town [7] and Fafan zone [2], with focus on employed and non-employed women in Ethiopia, the rate of EBF among employed women was 21% and 24.8%, respectively. Another cross-sectional study conducted in Dukem, central Ethiopia in 2015 showed that only 24.3% employed mothers exclusively breastfed their infants until six months [8].

Different factors are responsible for the low rate of EBF among employed and non-employed women. Good knowledge and positive attitude of mothers about the benefits of EBF plays an important role in its continuation until six months [9, 10]. A study in Jordan that showed support and encouragement from husbands and extended family members was associated with the increased rate of EBF for six months [11], whereas mother’s return to paid employment negatively impacted the duration of EBF [7, 12, 13]. Employed women need to return to work before six months because paid employment is a necessity, not an option for many of them [14]. Studies conducted among employed women shows different work-related factors that affect the continuation EBF either positively or negatively [3, 12, 13]. Availability of physical facilities and a supportive work environment such as flexibility and having supportive managers encourages women to continue EBF [3, 15].

Kebede T. et al reported several factors that triggered EBF discontinuation including a short duration of maternal leave, full-time employment, working in private organizations, lack of flexible working hours unable to express breast milk, lack of breaks to express breast milk and the workplace being far away from the child [8]. This study provides useful insights into some of the barriers to EBF for employed women in Ethiopia; however, the findings may not be generalisable to other areas of Ethiopia, as a very diverse country with different ethnicities and cultural expectations. Hence, the results of a study conducted in central Ethiopia, for example, may not be generalizable to people who live in the northern part of the country. Moreover, maternity leave in Ethiopia was increased from three to four months in 2018. The current study aimed to assess EBF continuation and associated factors among employed women in North Ethiopia, since the introduction of increased maternity leave. Knowledge of mothers about the benefits of EBF and its practice of EBF was also assessed.

## 2. Materials and methods

This study was part of a larger mixed-methods study looking at determinants of EBF among employed women after they returned to work. This article reports on survey results of the study. Findings are reported based on the Strengthening the Reporting of Observational Studies in Epidemiology Guidelines [16].

### 2.1 Study design and setting

This community-based, cross-sectional study was conducted in two towns in Tigray region, North Ethiopia between December 2018 and January 2019.

### 2.2 Participants

The study participants were full-time employed women who had children aged between six months and two years. Women working on contract, in casual, part-time or in their own business were excluded as they might have more flexible schedules.

### 2.3 Sampling method

**Fig 1:**
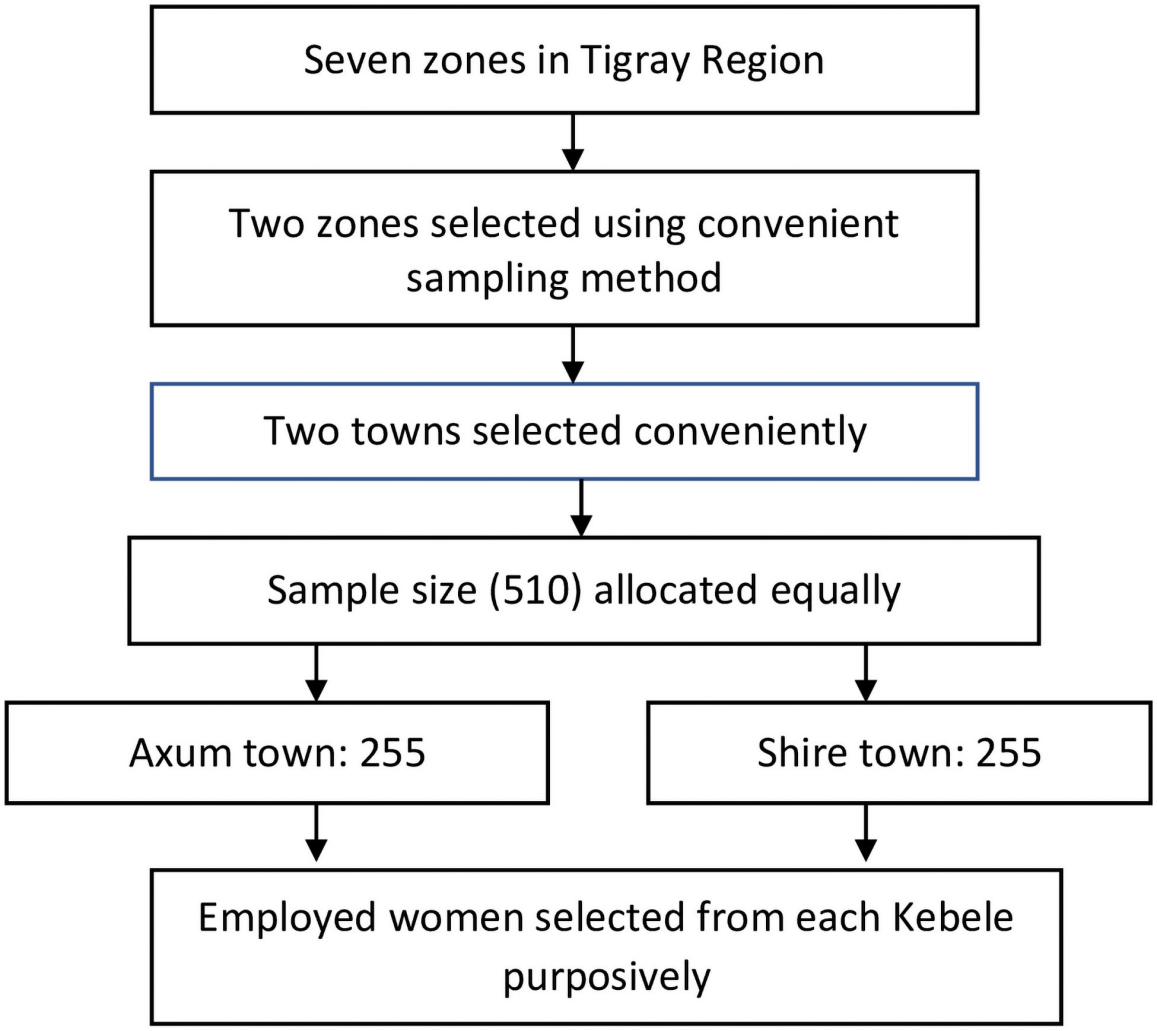
Sampling method of the study.

Multistage sampling was used to reach the study participants. First, two zones from the seven zones in the Tigray region were selected using a convenience sampling method. The biggest town was then selected from each zone because these towns are administrative and business centres in which many employed women live. All kebeles (lowest administrative division) in each town were included in the study. The total number of participants was allocated equally for each town. Finally, purposive sampling was used to recruit women from each kebele (Fig 1).

Twenty health extension workers from both towns visited women in their home. Potential participants were invited to the study and given the explanatory statement. Those who agreed to take part in the study were provided with a hard copy, self-administered questionnaire. It was explained that the questionnaire would be collected two weeks later. The first author supervised the overall data collection process.

### 2.4 Measurement of variables

We used a survey tool adapted from the Breastfeeding and Employment Study toolkit (BESt), developed by the University of Wisconsin [17]. The tool was modified to capture local factors deemed important. In total, 11 maternal characteristics and six family support related questions were added. The questionnaire comprises a total of 66 questions, 36 of which were four-point (strongly agree, agree, disagree and strongly disagree) Likert scale questions. The questionnaire was prepared in English and translated to local language (Tigrigna). Once participants completed the questionnaire it was back translated to English for data processing and analysis. Prior to distribution, the questionnaire was piloted on five employed mothers to ensure the appropriate wording and understandability by local Tigran mothers; no amendment was necessary.

The women were asked for how long they exclusively breastfed their last child. There were five alternatives to respond to this outcome variable (not at all, three months or less, four to six months, six months and more than six months). The independent variables were grouped into four 1) demographic characteristics, age of infants and mothers, marital status, type of work, educational status, monthly salary of mothers, number of children, place and type of birth; 2) EBF practice and knowledge about the benefits of EBF and breast milk expression; 3) family support that focused on the support mothers obtained at home from their husband and extended family members; and 4) work-related factors. Organisational managers support, co-workers support as well as time and physical environment related questions were addressed under the work-related factors.

### 2.5 Study size

The sample size was calculated using G*power with the following parameters: a power of 0.80, true proportion of estimated prevalence of EBF among employed women (21%) based on a previous study conducted in Gondar, Ethiopia [7] and a level of significance of 0.05 and sample size was 507.

### 2.6 Statistical analysis

We summarized continuous and categorical variables using mean (± standard deviations, SDs) and frequencies (percentages), respectively. The outcome variable (duration of EBF) was recoded as either yes (breastfed for six months or more) or no (Not at all, three months or less, four to six months breastfed) for the question “did you EBF your infant for six months”. The four-category responses (strongly agree, agree, disagree and strongly disagree) were collapsed into two categories (strongly agree/agree and strongly disagree/disagree).

Logistic regression analysis was used to assess the relationship between dependant and independent variables at 95% confidence interval. Initially all completed variables (n = 440) were included in the univariable logistic regression analysis. Variables with P-value <0.1 on the univariable logistic regression were included in the multivariable logistic regression. Variables with p-value 0.05 or less were considered statistically significant. SPSS software version 26 was used for the analyses.

### 2.7 Ethics approval and consent to participate

Ethical approval was obtained from Monash University Human Research Ethics Committee (ethics approval number: 13794) and Mekelle University Research Ethics Approval Committee (ethics approval number: ERC 1490/2018). Participation was voluntary and informed consent was obtained from each study participant prior to distribution of the questionnaire. To ensure their privacy, no personal identifiers of participants was used.

## 3. Results

Of the 510 questionnaires distributed, 449 (88.0%) were completed with equal number of completed questionnaires returned from each town. Nine out of 449 questionnaires were incomplete for the work-related section, and one out of nine questionnaires was incomplete for all sections except for demographic characteristics. Incomplete questionnaires were included in the descriptive statistics, but not in the logistic regression analysis.

### 3.1 Demographic characteristics of participants

The mean (SD) age of study participants was 30.4 (4.2) years. Three-hundred and seventy-nine (84.4%) participants were married and 337 (75.1%) had completed their education at diploma level or above. Three hundred and twenty-four (72.2%) respondents had either two or more live children, and 140 (31.2%) had monthly income of 107 USD or more. Four-hundred and forty-one (98.2%) mothers gave birth to their last child in a health facility and 303 (67.5%) had a spontaneous vaginal birth (Table 1).

**Table 1:** Demographic characteristics of study participants (n = 449).

Question/variable	n (%)
Age (years), mean (SD)	30.4 (4.2)
Age of youngest child (months), mean (SD)	12.1 (4.6)
**Marital status**	
Partnered	379 (84.4)
Unpartnered (single, divorced, widowed)	70 (15.6)
**Educational status**	
Secondary school or less	112 (24.9)
Diploma and above	337 (75.1)
**Type of work**	
Professional/skill	207 (46.1)
Administrative	186 (41.4)
Other	56 (12.5)
**Monthly salary (USD)**	
Less than 46	112 (25.2)
46–76	97 (21.6)
77–107	100 (22.3)
More than 107	140 (31.2)
**Number of live children per participant**	
One	125 (27.8)
Two or more	324 (72.2)
**Place of last birth**	
Home	8 (1.8)
Health center*	124 (27.6)
Hospital	317 (70.6)
**Mode of birth**	
Spontaneous vaginal birth	303 (67.5)
Instrumental assisted vaginal birth	48 (10.7)
Caesarean section	98 (21.8)

*Health centres are primary health care units that provide preventive and curative services with inpatient capacity of five beds.

Values are n (%) unless otherwise specified.

### 3.2 Breastfeeding practices and knowledge

Four-hundred and forty-eight participants responded to the section of questionnaire investigating breast feeding practices and knowledge. Of these women, 393 (87.7%) commenced breastfeeding within one hour of birth and 254 (56.6%) mothers exclusively breastfeed their children for six months or more. For 141 (31.5%) mothers who did not EBF, their primary reason for the introduction of additional food/fluids was the requirement to return to paid employment within six months of birth.

Four-hundred and forty (98.2%) participants believed that EBF has benefits. The common reasons identified as motivation for continuing EBF included nutritional benefits (58.0%), disease prevention (66.3%) and growth and development of infants (63.8). Contraceptive effect was another benefit of EBF mentioned by 350 (78.1%) of the study participants. A total of 293 (65.4%) respondents reported that they had information about expressed breast milk feeding. Health extension workers and professionals were the main sources of information about expressing breast milk for 156 (34.8%) and 158 (35.3%) mothers, respectively (Table 2).

**Table 2:** Breastfeeding practices and knowledge of study participants (n = 448).

Question/variable	n (%)
Started breastfeeding within one hour after birth	
Yes	393 (87.7)
No	55 (12.3)
Duration of EBF	
Six months or more	254 (56.6)
Less than six months	194 (43.4)
Reasons given for not adhering to EBF	
Belief that breast milk alone was not enough	38 (8.5)
Didn’t have enough milk	26 (5.8)
Started paid employment	141 (31.5)
Influence from family	3 (0.7)
Other	2 (0.5)
Belief that EBF is beneficial	
Yes	440 (98.2)
No	8 (1.8)
Mothers’ perceptions of benefits EBF for the infant**	
Nutritional benefits	260 (58.0)
Reduces risk of some diseases	297 (66.3)
Growth and development	286 (63.8)
Bonding between mother & infant	183 (40.8)
Mothers’ perceptions of benefits of EBF for herself/women**	
Contraceptive use	350 (78.1)
Control bleeding after birth	115 (25.7)
Decrease risk of breast/cervical cancer	112 (25.0)
Economic benefits	162 (36.2)
Awareness of how to express breast milk	
Yes	293 (65.4)
No	155 (34.6)
Source of information about expressed breast milk feeding**	
Health extension workers	156 (34.8)
Health professionals	158 (35.3)
Mass media (radio, TV etc.)	77 (17.2)
Social media	30 (6.7)
Other sources*	5 (1.1)
Mother fed her baby using expressed breastmilk	
Yes	109 (24.3)
No	339 (75.7)
Reason for expressed breastmilk feeding	
Returned to paid employment before six months	74 (16.5)
Unable to breastfeed after birth	23 (5.1)
Other	12 (2.7)

* other sources = individual woman’s knowledge as a health professional, family

**possible to give more than one answer

### 3.3 Family support of EBF

From the total study participants who responded (448), 371 (82.8%) reported that they received support from their family at home to continue EBF. The family members most commonly involved in supporting women to EBF were their husbands, their mothers and mothers-in-law as stated by 254 (56.7%), 172 (38.4%) and 61 (13.6%) participants, respectively. The participants also reported that 266 (59.4%) of husbands actively encouraged EBF. The common types of support women obtained at home were baby care 224 (50.0%) and staying with baby at home while they were at work 248 (55.4%). When mothers did not have support from their husband or family members, some would leave their infants with domestic workers at home 149 (33.3%) or take them to work 122 (27.2%) (Table 3).

**Table 3:** Family support of EBF among study participants (n = 448).

Question/variable	n (%)
Family support to continue EBF following return to paid employment	
Yes	371 (82.8)
No	77 (17.2)
Members of family who provided support at home	
Husband	254 (56.7)
Mother	172 (38.4)
Mother-in-law	61 (13.6)
Domestic worker	26 (5.8)
Other*	33 (7.4)
How do you rate the support you obtained from your husband?	
Unsupportive	59 (13.2)
Actively supportive	266 (59.4)
Supportive on request	93 (20.8)
Not applicable (no husband)	30 (6.7)
Type of support at home**	
No support	58 (12.9)
Baby care	224 (50.0)
Staying with baby at home	248 (55.4)
Household activities	150 (33.5)
Other	5 (1.1)
If no support at home, how you manage your child with work?	
Child minded at home by domestic worker	149 (33.3)
Day care (outside home)	24 (5.4)
Child taken to mother’s work	122 (27.2)
Other	4 (0.9)

*other: neighbours, extended family members

** possible to give more than one answer

### 3.4 Work-related factors affecting EBF

Four-hundred and forty participants identified a number of workplace factors that affected the continuation of EBF including receiving support from organizations, managers and co-workers, as well as availability of time and physical environment.

#### 3.4.1. Organizational support

In responding to the organizational support related questions, 277 (63.0%) participants agreed/strongly agreed that they wished they had enough maternity leave before going back to work. Three-hundred and fourteen (70.7%) participants disagreed/strongly disagreed that they had policies about breastfeeding in their workplace. One hundred and ninety-six (44.5%) participants strongly disagreed and a further 191 (43.4%) disagreed that they had access to an area at work, specifically designated for breastfeeding. From the participant mothers, 313 (71.1%) reported that their employment would not be at risk if they breastfeed in their workplace. A total of 306 (69.6%) participants disagreed/strongly disagreed that their opportunities for job advancement would be limited if they breastfeed at work (Table 4).

**Table 4:** Work-related factors affecting EBF (n = 440).

Variables	n (%)
**Organizational support**	
I would have enough (paid or unpaid) maternity leave to get breastfeeding started before going back to work.	
Strongly disagree/Disagree	163 (37.0)
Strongly agree/Agree	277 (63.0)
My company has written policies for employees that breastfeed or express breast milk.	
Strongly disagree/Disagree	314 (71.4)
Strongly agree/Agree	126 (28.6)
I would feel comfortable asking for space to breastfeed or express breast milk at work	
Strongly disagree/Disagree	357 (81.1)
Strongly agree/Agree	83 (18.9)
I’m certain there is a place I could go to breastfeed or express breast milk at work.	
Strongly disagree/Disagree	387 (88.0)
Strongly agree/Agree	53 (12.0)
There is someone at work that would help me plan for breastfeed or express breast milk	
Strongly disagree/Disagree	351 (79.8)
Strongly agree/Agree	89 (20.2)
My job could be at risk (e.g. lose my job) if I breastfed or express breast milk at work	
Strongly agree/Agree	316 (71.8)
Strongly disagree/Disagree	124 (28.2)
My opportunities for job advancement would be limited if I breastfed/express breast milk at work	
Strongly agree/Agree	306 (69.5)
Strongly disagree/Disagree	134 (30.5)
**Managers support**	
My manager would support me breastfeeding or expressing breast milk at work	
Strongly disagree/Disagree	320 (72.7)
Strongly agree/Agree	120 (27.3)
My manager would think I couldn’t finish all my work if I ask for a break for breastfeeding	
Strongly disagree/Disagree	293 (66.6)
Strongly agree/Agree	147 (33.4)
I would feel comfortable speaking with my manager about breastfeeding	
Strongly disagree/Disagree	282 (64.1)
Strongly agree/Agree	158 (35.9)
My manager would make sure my job is replaced if I need a break for breastfeeding or expressing breast milk	
Strongly disagree/Disagree	199 (45.2)
Strongly agree/Agree	241 (54.8)
My manager would change my work schedule to allow me time for breastfeeding or expressing breast milk	
Strongly disagree/Disagree	319 (72.5)
Strongly agree/Agree	121 (27.5)
My manager would help me deal with my workload to breastfeed/express breast milk	
Strongly disagree/Disagree	316 (71.8)
Strongly agree/Agree	124 (28.2)
**Co-workers’ support**	
I would feel comfortable speaking with my co-workers about breastfeeding	
Strongly disagree/Disagree	243 (55.2)
Strongly agree/Agree	197 (44.8)
My co-workers would change their break times so that I could breastfeed/express breast milk	
Strongly disagree/Disagree	188 (42.7)
Strongly agree/Agree	252 (57.3)
My co-workers would replace my job duties if I needed time for breastfeeding or expressing breast milk.	
Strongly disagree/Disagree	196 (44.5)
Strongly agree/Agree	244 (55.5)
**Time related variables and Physical environment**	
My breaks are frequent enough for breastfeeding or expressing breast milk.	
Strongly disagree/Disagree	358 (81.4)
Strongly agree/Agree	82 (18.6)
I could adjust my break schedule in order to breastfeed or express breast milk.	
Strongly disagree/Disagree	314 (71.4)
Strongly agree/Agree	126 (28.6)
I could buy or borrow the equipment I would need for expressing breast milk.	
No	356 (80.9)
Yes	84 (19.1)
My company would supply the equipment I need for expressing breast milk at work.	
No	417 (94.8)
Yes	23 (5.1)
There is a company-designated place for women to breastfeed or express milk	
No	440 (100)
Yes	0 (0)

#### 3.4.2. Managers’ support

Three hundred and twenty-three (73.4%) women disagreed/strongly disagreed that they had support from their managers to breastfeed at work. From the study participants, 293 (66.6%) mothers disagreed/strongly disagreed with the statement ‘my manager would think I couldn’t finish my work if I needed break for breastfeeding’. Two hundred and eighty-two (64.1%) mothers reported that they did not feel comfortable speaking about breastfeeding with managers. When talking about flexibility of the managers in supporting breastfeeding mothers, 241 (54.8%) participants reported that their managers want to make sure another person is available to undertake the work when the mothers needed time for breastfeeding. Ninety-one (20.7%) agreed and a further 30 (6.7%) strongly agreed, that their managers allowed them to change their work schedule for breastfeeding. However, 316 (71.8%) participants disclosed that their managers did not help them to manage their workload (Table 4).

#### 3.4.3. Co-workers support

From the participant mothers, 243 (55.3%) did not feel comfortable when speaking with co-workers about breastfeeding. However, 243 (55.3%) agreed/strongly agreed that their co-workers helped them by changing their break time to allow for breastfeeding or expressing breast milk. Similarly, 244 (55.4%) participants agreed/strongly agreed that their co-workers undertook their job to allow them time to breastfeed or express breast milk (Table 4).

#### 3.4.4. Time and physical environment

From the study participants, 80 (18.1%) agreed/strongly agreed that they had frequent enough breaks for breastfeeding or expressing breast milk. A total of 126 (28.6%) agreed/strongly agreed that they could adjust their schedule to make time for breastfeeding. Whereas, when talking about accessibility of equipment for breast milk expression, only 84 (19.1%) reported that they could buy or borrow equipment for expressing breast milk. All (100%) participants reported that none of the companies they work for had a designated place for women to breastfeed or express milk during the work day (Table 4).

### 3.5 EBF and associated factors among employed women

Two-hundred and fifty-four (56.6%) participants reported that they exclusively breastfed their infants until six months. The main reason for 46.4% of participants who did not adhere to EBF was returning to work before six months.

Of the variables used in the univariable logistic regression, only six variables had a p-value of <0.1 and were used in the multivariable logistic regression (Table 5). Mothers who had family support were two times more likely to continue EBF, compared to those who did not have family support (AOR = 2.1, 95%, CI 1.2–3.6; P = 0.005). Similarly, mothers who agreed/strongly agreed of having frequent enough breaks were 2.6 times more likely to EBF than those who disagreed/strongly disagreed (AOR = 2.6, 95% CI, 1.4–4.8; P = 0.002). When the mothers could buy or borrow equipment needed for expressing breast milk, they were 1.6 times more likely to continue EBF compared to those who could not (AOR = 1.7, 95% CI, 1.0–3.0; P = 0.033). Table 5 mainly presented the adjusted odds ratio of the variables that show statistical association. However, the crude odds ratio of some important variables is included on Table 5.

**Table 5:** Logistic regression for work-related predictors of EBF among employed women (n = 440).

Variables	n	Crude OR (95% CI); p values	Adjusted OR (95% CI); p values
Age of mother (years)			NI
18–29	265	Ref.	
30 or more	175	1.2 (0.8–1.9); 0.263	
Marital status			NI
Partnered	373	Ref.	
Unpartnered (single, divorced, widowed)	67	1.2 (0.7–2.1); 0.439	
Educational status			NI
Secondary or less	103	Ref.	
Diploma or more	337	1.3 (0.7–2.2); 0.304	
Monthly salary, (USD)			NI
76 or less	200	Ref.	
Greater than 76	240	0.8 (0.5–1.3); 0.518	
Number of children			NI
One	125	Ref.	
Two or more	315	0.7 (0.4–1.1); 0.166	
Awareness about breast milk expression			
No	151	Ref.	Ref
Yes	289	1.5 (1.0–2.3); 0.046	1.1 (0.7–1.7); 0.469
Family support to continue EBF following return to paid employment			
No	77	Ref.	Ref
Yes	363	2.3 (1.4–3.9); 0.001	2.1 (1.2–3.6); 0.009
I would have enough maternity leave (paid and/or unpaid) to get BF started before going back to work			
Strongly disagree/Disagree	163	Ref.	NI
Strongly agree/Agree	277	1.2(0.7–1.9); 0.351	
I’m certain there is a place I could go to breastfeed or express breast milk at work			
Strongly disagree/Disagree	387	Ref.	Ref
Strongly agree/Agree	53	0.4 (0.2–1.0); 0.081	0.7 (0.4–1.5); 0.455
I would feel comfortable asking for accommodation for breastfeeding or express breast milk at work.			
Strongly disagree/Disagree	357	Ref.	Ref.
Strongly agree/Agree	83	0.5 (0.2–1.1); 0.099	0.6 (0.3–1.2); 0.182
My manager would change my work schedule to allow me time to breastfeed			
Strongly disagree/Disagree	320	Ref.	Ref.
Strongly agree/Agree	120	0.4 (0.2–1.0); 0.062	0.7 (0.4–1.2); 0.282
My breaks are frequent enough for breastfeeding or expressing breast milk.			
Strongly disagree/Disagree	358	Ref.	Ref.
Strongly agree/Agree	82	2.5 (1.1–5.6); 0.018	2.6 (1.4–4.8); 0.002
I could buy or borrow the equipment I would need for expressing breast milk.			
No	356	Ref.	Ref
Yes	84	2.0 (1.1–3.8); 0.022	1.7 (1.0–3.0); 0.033

NI: Not included

## 4. Discussion

In this study more than half of the employed women reported EBF and the majority were aware of the benefits of EBF for infants and mothers and reported family support. While more than half of the participants agreed/strongly agreed that their co-workers helped them to change their work schedule, around three quarters of the participants disagreed/strongly disagreed that they had support from their managers. All participants reported that there is no designated place for women to breastfeed or express milk in their workplace. Over half of the study participants reported EBF at six months. Factors associated with EBF were having family support, having frequent breaks at work and the possibility of buying or borrowing equipment for expressing breast milk.

The prevalence of EBF in our study (56.4%) was higher compared to other studies conducted in other areas of Ethiopia including Gondar in 2015 (20.9%), Dukem in 2015 (24.3%) and Fafan in 2016 (24.8%) [2, 7, 8]. The reason for the increase in EBF might be a consequence of recent improvements in maternity leave in Ethiopia. Besides, women can use their annual leave after they finish their four months maternity leave which assists employed women to extend the duration of EBF at least by one month. Our prevalence of EBF was also higher compared to other reported prevalence in other areas of Africa such as Ghana (10.3%) and Egypt (14.1%) [18, 19]. The difference might be due to the difference in leave entitlements, or local cultural practices and social expectations.

Awareness of mothers about the benefits of EBF is crucial for its continuation. In this study 98.2% participants believe that EBF has benefits. A similar finding was obtained in a study conducted in Fafan (Somali region of Ethiopia) in which all participants were aware of the benefits of EBF [2]. Studies conducted in Ghana and South Jordan also found that 99% and 99.3% mothers were aware of the benefits of EBF, respectively [18, 20]. However, in studies conducted in Gondar, Ethiopia and Nigeria, 80% and 77.5% participants acknowledged the benefits of EBF, respectively [7, 21]. These figures suggested that employed women have good awareness of the benefits of EBF which could motivate them to continue EBF until six months, even after they have returned to work [7].

In this study, having family support was positively associated with continuation of EBF. Common supports mothers obtained at home include baby care, staying with baby at home while she was at work, helping with cooking and other household activities. A similar finding was obtained in a study conducted among working women in Indonesia [22]. The authors of the Indonesian study found that mothers who had family support were two times more likely than those who did not have the support to exclusively breastfeed [22]. However, a study conducted in Gondar, Ethiopia showed that mothers who had no family support were more likely to exclusively breastfeed as compared to those who had family support [7]. This was an unexpected finding not supported by the literature. Women who have support at home could spend more time with their infants which helped the children get adequate breastmilk. Therefore, having family support encouraged women to continue EBF after they returned to paid employment.

Participants who had frequent breaks at work were more likely to continue EBF. In this study mothers who agreed/strongly agreed that they had frequent enough breaks at work were more likely to continue EBF as compared to those who disagreed/strongly disagreed. Similarly, in the study conducted in Dukem, central Ethiopia [8], mothers who had no break time at work were more likely to discontinue EBF as compared to those who had breaks. This would be because having sufficient breaks might encourage women to continue EBF after they returned to work [23]. When employed women have breaks they could either go home to breastfeed their infants if their home is nearby or ask someone to bring their infants to work for breastfeeding.

Participants who could buy or borrow equipment they need for expressing breast milk, were more likely to continue EBF as compared to those who could not afford it. This means, if mothers have access to equipment to express their breast milk, they can exclusively breastfeed for longer. However, there is no existing literature to compare with this finding. Therefore, this could be a new finding in this study.

This study has limitations. Firstly, self-administered questionnaire was used to collect the data which makes it difficult for women to seek clarification for any question. Secondly, employed women with children up to two years were included in the study which could lead to recall bias of the exact duration of EBF. Thirdly, the study participants were government employees. Therefore, the findings would not represent women employed in private organizations. Further research with a focus on women employed in private organizations is recommended. Lastly, we used convenience sampling in northern Ethiopia and the findings might not be generalizable to all employed women in Ethiopia.

## 5. Conclusion

Almost all participants were aware of the benefits of breastfeeding. Our findings show increased percentage of EBF among employed women in Ethiopia compared to previous studies [2, 7, 8]. We tend to think that this might be partly contributed by the recent increase in maternity leave in Ethiopia because many of the children in this study were born after July 2018, when the policy changed. Although other factors play roles in EBF, based on the most common reason for EBF cessation in this study, we believe that six-month’s maternity leave might be the most helpful solution for working women to be able to exclusively breastfeed their baby. In the absence of six-month maternity leave, the role of families and managers’ role is of importance in continuing EBF is critical.

## Supporting information

S1 Data (SAV)

S1 Table Logistic regression for predictors of EBF among employed women in North Ethiopia. (n = 440). (DOCX)
