# Supplementary material for: Exclusive breastfeeding continuation and associated factors among employed women in North Ethiopia: A cross-sectional study
Source: PLoS One. 2021 Jul 29;16(7):e0252445. doi: 10.1371/journal.pone.0252445 (PMC8321127; doi:10.1371/journal.pone.0252445)
Supplement: S1 Table — (DOCX) [file pone.0252445.s002.docx]

**S1 Table :** **Logistic regression for predictors of** **EBF among employed women in North Ethiopia. (n=440)**

| **Variables** | **n** | **Crude OR (95% CI); p values** | **Adjusted OR (95% CI); p values** |
| --- | --- | --- | --- |
| Age of mother (in years)  18-30  31 or more | 265  175 | Ref.  1.2(0.7-1.8); 0.457 |  |
| Marital status  Married  Unpartnered | 373  67 | Ref.  1.2(0.7-2.1); 0.370 |  |
| Type of work  Professional  Non-professional | 207  233 | Ref.  0.8 (0.5-1.3); 0.460 |  |
| Educational Status  Secondary or less  Diploma or more | 102  338 | Ref.  1.4 (0.8-2.3); 0.229 |  |
| Monthly salary (Birr)  2500 or less (76 USD or less)  Greater than 2500 (greater than 76 USD) | 200  240 | Ref.  0.8 (0.5-1.3); 0.445 |  |
| Number of children  One  Two or more | 125  315 | Ref.  0.9(0.5-1.4); 0.645 |  |
| Place of last birth  Home  Health institution | 07  433 | Ref.  1.4(0.3-6.1); 0.657 |  |
| Mode of birth  Vaginal  Caesarean section | 343  97 | Ref.  0.9(0.6-1.5); 0.886 |  |
| Starting BF within 1 hour after birth  No  Yes | 54  386 | Ref.  0.7(0.4-1.3); 0.320 |  |
| Benefits of BF  No  Yes | 8  432 | Ref.  0.4(0.1-1.7); 0.219 |  |
| Awareness about breast milk expression  No  Yes | 151  289 | Ref.  1.5 (1.0-2.3); 0.046 | Ref  1.1 (0.7–1.7); 0.469 |
| Ever fed a baby with expressed breastmilk  No  Yes | 334  106 | Ref.  1.4(0.8-2.2); 0.148 |  |
| Family support to continue BF  No  Yes | 77  363 | Ref.  2.3 (1.4-3.9); 0.001 | Ref  2.1 (1.2-3.6); 0.009 |
| **Organizational support** |  |  |  |
| I would have enough maternity leave (paid and/or unpaid) to get BF started before going back to work.  Strongly disagree/Disagree  Strongly agree/Agree | 163  277 | Ref.  1.2(0.7-1.9); 0.351 |  |
| I would be able to get information about combining work and BF from my company.  Strongly disagree/Disagree  Strongly agree/Agree | 295  145 | Ref.  0.4(0.8-1.4); 0.464 |  |
| My company has written policies for employees that BF or pumping breast milk.  Strongly disagree/Disagree  Strongly agree/Agree | 314  126 | Ref.  0.9(0.5-1.7); 0.926 |  |
| I’m certain there is a place I could go to breastfeed or pump breast milk at work.  Strongly disagree/Disagree  Strongly agree/Agree | 387  53 | Ref.  0.4 (0.2-1.0); 0.081 | Ref  0.7 (0.4–1.5); 0.455 |
| There is someone at work that would help me make arrangements for BF or pumping breast milk  Strongly disagree/Disagree  Strongly agree/Agree | 351  89 | Ref.  0.6(0.3-1.3); 0.214 |  |
| My job could be at risk (e.g. lose my job) if I breastfed or pumped breast milk at work.  Strongly agree/Agree  Strongly disagree/Disagree | 316  124 | Ref.  1.2(0.7-2.0); 0.447 |  |
| I would be able to talk about BF at work  Strongly disagree/Disagree  Strongly agree/Agree | 236  204 | Ref.  1.0(0.6-1.7); 0.786 |  |
| I would feel comfortable asking for accommodations to help me breastfeed or pump breast milk at work.  Strongly disagree/Disagree  Strongly agree/Agree | 357  83 | Ref.  0.5 (0.2-1.1); 0.099 | Ref.  0.6 (0.3-1.2); 0.182 |
| My opportunities for job advancement would be limited if I breastfed or pumped breast milk at work.  Strongly agree/Agree  Strongly disagree/Disagree | 306  134 | Ref.  1.3(0.7-2.4); 0.264 |  |
| I’m certain that women in higher-level positions have breastfed milk at my workplace.  Strongly disagree/Disagree  Strongly agree/Agree | 327  113 | Ref.  0.8(0.4-1.5); 0.537 |  |
| I’m certain coworkers have breastfed or pumped breast milk at my workplace.  Strongly disagree/Disagree  Strongly agree/Agree | 317  123 | Ref.  0.6(0.3-1.1); 0.130 |  |
| **Managers support** |  |  |  |
| My manager would support me BF/pumping breast milk at work  Strongly disagree/Disagree  Strongly agree/Agree | 320  120 | Ref.  0.7(0.3-1.8); 0.525 |  |
| My manager would help me combine breastfeeding and work.  Strongly disagree/Disagree  Strongly agree/Agree | 327  113 | Ref.  1.5(0.6-3.6); 0.381 |  |
| My manager would think I couldn’t get all my work done if I needed to take breaks for BF  Strongly disagree/Disagree  Strongly agree/Agree | 293  147 | Ref.  1.3 (0.8-2.2); 0.229 |  |
| I would feel comfortable speaking with my manager about BF.  Strongly disagree/Disagree  Strongly agree/Agree | 282  158 | Ref.  0.6(0.3-1.1); 0.153 |  |
| My manager says things that make me think he/she supports Bf  Strongly disagree/Disagree  Strongly agree/Agree | 299  141 | Ref.  0.8(0.4-1.5); 0.556 |  |
| I feel my manager would view BF as an employee’s personal choice.  Strongly disagree/Disagree  Strongly agree/Agree | 259  181 | Ref.  1.1(0.6-1.8); 0.666 |  |
| My manager would consider it part of his/her job to help me combine BF and work.  Strongly disagree/Disagree  Strongly agree/Agree | 324  116 | Ref.  0.7(0.4-1.4); 0.390 |  |
| My manager would make sure my job is covered if I needed time for BF or pumping breast milk  Strongly disagree/Disagree  Strongly agree/Agree | 199  241 | Ref.  0.9(0.5-1.5); 0.723 |  |
| My manager would change my work schedule to allow me time for BF or pumping breast milk  Strongly disagree/Disagree  Strongly agree/Agree | 319  121 | Ref.  0.4 (0.2-1.0); 0.062 | Ref.  0.7 (0.4-1.2); 0.282 |
| My manager would help me deal with my workload, so I could breastfeed or pump breast milk at work.  Strongly disagree/Disagree  Strongly agree/Agree | 316  124 | Ref.  0.7(0.3-1.4); 0.360 |  |
| My manager would be embarrassed if I spoke with him/her about BF.  Strongly agree/Agree  Strongly disagree/Disagree | 334  106 | Ref.  0.9(0.5-1.6); 0.781 |  |
| **Co-workers support** |  |  |  |
| My co-workers would think less of workers that choose to breastfeed or pump breast milk at work.  Strongly disagree/Disagree  Strongly agree/Agree | 278  162 | Ref.  0.8(0.5-1.4); 0.541 |  |
| I would feel comfortable speaking with my co-workers about BF  Strongly disagree/Disagree  Strongly agree/Agree | 243  197 | Ref.  1.4(0.8-2.6); 0.175 |  |
| My co-workers say things that make me think they support BF.  Strongly disagree/Disagree  Strongly agree/Agree | 237  203 | Ref.  0.9(0.5-1.7); 0.919 |  |
| My co-workers would change their break times with me so that I could breastfeed or pump breast milk  Strongly disagree/Disagree  Strongly agree/Agree | 188  252 | Ref.  1.0(0.4-2.2); 0.892 |  |
| My co-workers would cover my job duties if I needed time for BF or pumping breast milk.  Strongly disagree/Disagree  Strongly agree/Agree | 196  244 | Ref.  0.9(0.4-1.8); 0.810 |  |
| My co-workers would be embarrassed if I spoke about BF.  Strongly agree/Agree  Strongly disagree/Disagree | 342  98 | Ref.  1.2(0.7-2.1); 0.378 |  |
| **Time related variables** |  |  |  |
| My breaks are frequent enough for BF or pumping breast milk.  Strongly disagree/Disagree  Strongly agree/Agree | 358  82 | Ref.  2.5 (1.1-5.6); 0.018 | Ref.  2.6 (1.4-4.8); 0.002 |
| My breaks are long enough for BF or pumping breast milk.  Strongly disagree/Disagree  Strongly agree/Agree | 405  35 | Ref.  1.3(0.5-3.5); 0.541 |  |
| I could adjust my break schedule in order to breastfeed/pump breast milk.  Strongly disagree/Disagree  Strongly agree/Agree | 314  126 | Ref.  1.1(0.6-2.2); 0.617 |  |
| **Physical environment** |  |  |  |
| I could buy or borrow the equipment I would need for pumping breast milk.  No  Yes | 356  84 | Ref.  2.0 (1.1-3.8); 0.022 | Ref  1.7 (1.0-3.0); 0.033 |
| My company would supply the equipment I would need for pumping breast milk at work  No  Yes | 417  23 | Ref.  0.5(0.1-1.8); 0.341 |  |
| I could find a place to store expressed breast milk at work.  No  Yes | 428  12 | Ref.  0.7(0.1-2.8); 0.647 |  |
