# Supplementary material for: Exclusive breastfeeding continuation and associated factors among employed women in North Ethiopia: A cross-sectional study
Source: PLoS One. 2021 Jul 29;16(7):e0252445. doi: 10.1371/journal.pone.0252445 (PMC8321127; doi:10.1371/journal.pone.0252445)
Supplement: Attachment — Submitted filename: Response to Reviewer V2.docx [file pone.0252445.s003.docx]

**PONE-D-20-37978**

**Exclusive breastfeeding continuation and associated factors among employed women in North Ethiopia: A cross-sectional study**

Dear Dr Frank T. Spradley,

The Editor PLOS ONE

Thank you for considering our manuscript for publication in the “PLOS ONE” Journal.

We have addressed the editor and reviewers’ helpful comments point by point as follows.

Kind regards,

Kahsu Gebrekidan, on behalf of all authors

**Reviewer #1:**

This is a very important and interesting study so I'm keen to see it published. I am thanking the authors for their hard work and hope that my comments below will be useful for improving the manuscript.

- **Response:** Thank you very much.

**Major comments and/or request for clarifications:**

1. P.7, line 125-129: Sample size. This manuscript was looking at the prevalence among EBF among employed women. In this case, I think the authors should have been using an estimated prevalence of EBF among employed women (from previous studies) as the reference to calculate their sample, instead of prevalence of employed women in the community.

- **Response:** Thank you for your comment, it was a typographical error now corrected on page 7, line 127. The sample size was calculated using estimated prevalence of EBF among employed women obtained from a study conducted in Gondar, Ethiopia (ref. 7).
  - This also brought further comment to the result and discussion. The ORs that they got from multivariable analysis ranged from 0.6-2.6, so for some variables, and I am worried that their sample may not be enough. If this is a limitation, it may be helpful to mention it in the discussion. Otherwise, I would be happy to receive any clarification.
    - **Response:** Thank you for your concern. Please note that the CI of 0.6- 2.6 is not considered wide. Our sample size was for the logistic regression was based on the rule of thumb for sample size in regression analysis is that for regression equations using six or more predictors, an absolute minimum of 10 participants per predictor variable is appropriate. (ref: Harris, R. J. (1985). A primer of multivariate statistics (2nd ed.). New York: Academic Press).

2. P7, line 130-141. Statistical analysis. I believe that the authors have carefully considered the statistical approach to measure effect size. However, given the high prevalence of outcome in this study population, I wonder why the authors chose logistic regression. I am aware that OR is widely used to approximate RR as the ‘true’ effect size in studies that investigate associated factors. However, ORs produced by logistic regression in a sample with prevalent outcome tend to be overestimated. Apologies if I missed it, but it was not mentioned in the discussion and I was struggling to find the authors’ justification for not using other statistical approach such as modified Poisson regression to estimate effect size. I hope these can be useful references (Barros and Hirakata, 2003, [https://pubmed.ncbi.nlm.nih.gov/14567763/and Zou,2004](https://pubmed.ncbi.nlm.nih.gov/14567763/and%20Zou,2004), <https://academic.oup.com/aje/article/159/7/702/71883>).

- **Response:** Thank you very much for recommending the helpful references and for the suggestion. While your point is valid, however, logistic regression analysis for binary outcomes is frequently used in epidemiological studies.

3. Coherence between introduction and conclusion. In line 309-310, the authors stated, “Our findings show a reassuring increased percentage of EBF among employed women which might be due to a recent increase in maternity leave in Ethiopia”. According to the introduction in line 78, policy change of maternity leave in Ethiopia (increased length from three to four months) took place in2018. This study was done later in December 2018 – January 2019 (line 89) and the study participants were women whose child was six-month-old up to two-year-old. If this study was to assess EBF continuation and associated factors among employed women since the introduction of increased maternal leave (line 80), then to my understanding, this timeline could not support authors’ conclusion.
   - **Response:** Thank you for your comment. The increment of the maternity leave was started on 1^st^ July 2018 and line 78 has been corrected to reflect this.
4. Table 2. Reasons for not continuing EBF. I wonder if the participants can only pick one. Out of curiosity, I would love to hear further explanation on the situation where someone had two or more reasons.
   - **Response:** Thank you for your comment. On the questionnaire, it was stated that the participants could provide more than one answer, an alternative there was “others (specify)”. As a result, 16 of 210 participants responded more than one answer.
5. Table 2. Some women cannot breastfeed because of medical conditions. I did not see medical or health-related reasons being mentioned or discussed in this study. Was this condition an exclusion criterion, was it not asked, or none of the participants experienced it?
   - **Response:** We agree that medical condition could be a factor for EBF discontinuation. In our study it was possible to give a related answer under the response “if others (please specify)” but none of the participants experienced it.
6. Table 3. In row 2, domestic worker was listed as part of family support, but in row 5, it was asked as an option for participants who did not have support at home. This is a bit confusing and I hope the authors can clarify.
   - **Response:** Thank you for your comment. Some participants mentioned that the domestic workers were part of their extended family members and responded under family support. This category was added during data coding because many participants responded this under the category “if others (please specify)”. For other participants the domestic workers were not considered as part of their extended family.
7. Table 4. Question “I would have enough (paid or unpaid) maternity leave to get breastfeeding started before going back to work”. In line 198, it meant the participants felt that they had enough maternity leave. I would rephrase the question so that it would be clearer. I initially was not sure whether that statement meant the participants had enough maternity leave or it was something that they wished they had/desired in the future.
   - **Response:** Thank you for your comment. The statement has been rephrased to include the recommended changes on page 12 line 199
8. Table 5. I wonder how the authors selected the variables to include in their multivariable analysis. I read that only complete question was included, but is there any other consideration? What about other important variables, e.g. having enough maternity leave? In my opinion, this variable is highly relevant to the study objective. However, I did not manage to find the reason it was not included while some other work-related variables (e.g. certainty about place to express breastmilk at work) were.
   - **Response:** Thank you for your comment. Initially all completed variables (n=440) were included in the univariable logistic regression analysis. As a result, all the work-related variables were included. Some variables from part 2: breastfeeding knowledge and practice and part 3: family support to EBF were excluded. Variables with P-value <0.1 in the univariable logistic regression were included in the multivariable logistic regression.
9. Table 5. I observe a major flip between all the crude ORs and adjusted ORs. Was the reference category for the crude ORs different from that for adjusted ORs?
   - **Response:** Thank you for your feedback, the reference category for the COR has been reversed, now it is amended (Table 5 page 17)
10. Line 313. The authors stated, “Maternal leave for six months is the best solution for women to adhere to EBF”. As much as I personally want this policy to come true, I struggled to find which part of the findings that supported this conclusion.
    - **Response:** Thank you for your comment. In our study the main reason many mothers discontinue EBF was returning to work before six months (page 10 line 170). That is why maternal leave for six months is the best solution for women to adhere to EBF.

**Minor comments:**

1. P2, line 26 and elsewhere: terminology. To my knowledge, the official term is “convenience sampling” instead of “convenient sampling” (Sedgwick, 2013, <https://www.bmj.com/content/347/bmj.f6304>).

- **Response:** Thank you for your feedback, all references to convenient sampling have been changed to convenience sampling on pages 2, 6 and 20

2. P2, line 30 and elsewhere: terminology; multivariable instead multivariate (and likewise, P7 line 138 and elsewhere: univariable instead of univariate). To my understanding, multivariate means multiple outcomes (dependent variables) and multivariable means multiple independent variables (Hidalgo and Goodman, 2013, <https://www.ncbi.nlm.nih.gov/pmc/articles/PMC3518362/>). I am aware that there has been a debate on this terminology over the years, so I am open for other arguments/insights either from the authors, other reviewers, or the editors.

- **Response:** Thank you for your comment. The recommended changes are done on pages 2, 7, 16: univariate changed to univariable and multivariate changed to multivariable.
